# Selective dorsal rhizotomy long-term effects on functional motility in Jordanian children with spastic cerebral palsy

**DOI:** 10.3389/fneur.2025.1502451

**Published:** 2025-01-29

**Authors:** Yazan E. Al-kharabsheh, Anas Said, Ismail A. Ismaiel, Issam Khawaja, Marwan Altaher, Ali Bani-Ahmed, Carmen M. Cirstea

**Affiliations:** ^1^University of Missouri School of Medicine, Columbia, MO, United States; ^2^Department of Neurosurgery, Al Bashir Hospital, Amman, Jordan; ^3^Department of Rehabilitation Sciences, Jordan University of Science and Technology, Irbid, Jordan; ^4^Department of Physical Medicine and Rehabilitation, University of Missouri School of Medicine, Columbia, MO, United States

**Keywords:** spastic cerebral palsy, selective dorsal rhizotomy, Jordan, functional mobility, children

## Abstract

**Introduction:**

Spasticity management in children with cerebral palsy (CP) is a challenge for healthcare providers worldwide. In the US and Europe, treatment options include non-surgical and surgical (i.e., selective dorsal rhizotomy, SDR) procedures, with beneficial effects on functional motility. SDR was introduced in Jordan in 2019. We performed the first assessment of the long-term effects on motor function in Jordanian children with spastic CP (SCP) who underwent SDR.

**Methods:**

A retrospective cohort study of 43 patients (28 boys, 15 girls, mean ± SD age at surgery, 6.2 ± 2.5 years, 67.4% with diplegia, 30.2% quadriplegia, and 2.3% hemiplegia, 97.7% bilateral deficits) who received SDR (42 bilateral) was conducted between 01/01/2019 and 03/01/2023. Gross Motor Function Classification System (GMFCS) and Functional Mobility Scale (FMS) scores were compared before and 12 months after SDR. Sex, age and clinical scores at surgery, and post-SDR surgical treatment were included in the model (IBM SPSS Statistics 29.0).

**Results:**

Clinical scores improved 12 months after SDR: GMFCS decreased by at least one level (in 58.5% of patients), and FMS significantly increased (*p* < 0.001); GMFCS decreased in 77.7% of those with pre-SDR severe impairment vs. 43.5% in moderately to mildly impaired patients. An age sub-analysis demonstrated higher changes in GMFCS in younger children (GMFCS decreased in 46.9% of those aged <10 years old vs. none in those older than 10 years). These findings suggest that younger children (<10 years old) and more impaired (levels IV and V on GMFCS) are likely the best candidates for this procedure. Twelve-month functional improvement was similar in boys and girls (GMFCS decreased in 44.0% of boys vs. 37.5% of girls). Compared to pre-SDR management, all patients continued physiotherapy, less received Botox (by 97.7%), and more received adjunct orthopedic surgeries (32.6% vs. none) after SDR; out of those receiving post-SDR adjuvant surgeries, 50.0% improved GMFCS (compared to 64.0% of those without).

**Conclusion:**

Our data demonstrated SDR’s beneficial long-term effects on functional mobility in SCP children, particularly those younger than 10 years and more severely impaired. These findings provide critical information that may aid in identifying “the best” therapeutic window and “the best” candidate for SDR in Jordan.

## 1 Introduction

Cerebral palsy (CP) is a non-progressive, permanent neuromotor disorder resulting from damage to the developing brain in the perinatal period (mostly clinically detectable in the first 2 years of life) with an incidence of 1 out of 500 births (1, 2). Major risk factors for CP are prematurity, low birth weight, perinatal complications (e.g., chorioamnionitis, birth-related intracerebral hemorrhage), postnatal diseases (e.g., meningitis), or trauma. There are several types of CP: spastic, dyskinetic, hypotonic, and mixed. This study focused on patients with spastic CP (SCP), the most common CP clinical phenotype affecting 80% of patients (2–4). Physical indicators of SCP include spasticity affecting multiple muscles, more than one limb, scissors gait, and persistence of primitive reflexes; all these impairments result in functional disturbances with daily living activities, even severe physical disability (3). Spasticity management in SCP is a major challenge for healthcare professionals worldwide. Various forms of spasticity treatments are available: physical therapy, occupational therapy, oral or intrathecal medications, orthopedic surgery, and selective dorsal rhizotomy (SDR) (5).

SDR is a neurosurgical procedure sectioning the lumbosacral afferent nerve rootlets, showing a significant reduction of the spasticity associated with SCP (6, 7). The initial applicability of this procedure was to alleviate pain more than a century ago. It was not until the early 1900s that SDR was first used to treat spasticity (8); since then, the safety and efficacy of this procedure have been refined, and SDR is now implemented globally as a treatment option for spasticity in children with SCP (8). This procedure has recently been used for SCP in Jordan, with the first procedure ever done in 2019 at the Al Bashir Hospital, Amman. There are no studies on the long-term efficacy of SDR in Jordanian children.

Here, we propose a retrospective study aimed at gathering, evidence of SDR’s long-term effects on functional outcomes in these children. We hope the findings of this study will make neurologists and neurosurgeons outside of Amman more aware of the benefits of this procedure and, more importantly, help counsel patients and their families about expected outcomes after such a procedure.

## 2 Materials and methods

Appropriate University of Missouri—Columbia and Al Bashir Hospital Institutional Review Board approvals were obtained for this study. Data of SCP patients aged 3–15 years old, who underwent SDR surgery between July 1, 2019, and June 30, 2023, were extracted from the Al Bashir Hospital’s medical records. Any patient 16 years old or older and 3 years of age or younger were excluded from the study. Demographic information (sex, age), clinical data, and treatment history (pre or baseline and post-SDR) were collected for each participant. This hospital is the only hospital recognized in Jordan for SDR treatment in SCP patients; candidates selected for this procedure usually have significant spasticity associated with a critical reduction in mobility.

Briefly, for an SDR procedure, the patient is placed in a prone position to gain access to the lumbar spine. Lumbosacral MRI is obtained pre-operatively to localize the conus and identify the level of the laminectomy. A 1–2-inch incision is made at the vertebral level between L1 to L5 along the midline of the lumbar spine based on conus location. To expose the spinal cord and rootlets, a high-speed electrical saw (Midas) is used for long-segment laminotomy. Surgical microscope identification is utilized to identify the natural separation between sensory and motor nerves. A rubber pad is then used to separate the two nerve groups to isolate sensory rootlets for the procedure; the motor nerves are separated away from the operative field. The exposed sensory nerve roots are each divided into multiple rootlets for electrical pattern measurement. Using EMG, the rootlets are tested for conduction abnormalities to determine which rootlets to section, minimizing spasticity and sensory loss. Rootlets are graded based on the severity of abnormal conduction from 0 to 4, the latter being the most severe. Rootlets that are deemed to have a severe rating are ligated. This process is performed for sensory nerve roots between L1 and S2.

Clinical data included in this study were the Patient Gross Motor Function Classification System (GMFCS), Functional Mobility Scale (FMS), and Australian Spasticity Assessment Scale (ASAS) scores before and at 12 months after SDR. The 12-month changes in scores of GMFCS and FMS were used to detect the long-term effects of the SDR procedure; changes in ASAS were discussed here as secondary outcomes. GMFCS is a standardized tool used to classify gross motor function in patients with cerebral palsy; this scale consists of five levels, from level one, reflecting limitations in fine motor skills, like coordination, balance, and speed, to level five, when patients are transported in a manual wheelchair in all settings and are limited in posture and limb movement (9). FMS is another standardized tool used to classify gross motor function, consisting of six levels (10). At level one, a wheelchair user may stand or transfer and may do some stepping supported by another person or using a walker. In contrast, at level six, patients are independent on all surfaces, do not use aids, do not need another’s assistance, and can walk over uneven or crowded surfaces. ASAS is also a standardized tool used to quantify the spasticity of hip flexors, quadriceps, hamstring, gastrocnemius & soleus, and tibialis (in this study), consisting of five levels scored from 0 (no spasticity) to 4 (severe spasticity) (11).

A two-sample paired *t*-test (or Wilcoxon signed-rank test) was used to compare changes in GMFCS, FMS, and ASAS values from baseline, before SDR, with a 12-month timepoint. Pearson correlation analysis was used to determine if the changes in FMS (Δ = 12-month score − baseline score) are impacted by the age at surgery; note that skewness values for ΔFMS was 0.4 (value generally acceptable for normal distribution). Data were reported as mean ± standard deviation, median (25th–75th percentile), and percentage (%) of the entire sample (*n* = 41 for GMFCS and *n* = 42 for FMS). Subgroup analyses (based on age, sex, and baseline clinical impairment) were also performed. ASAS changes between baseline and 12 months were presented as % change considering the baseline impairment level, i.e., % change ASAS = (mean 12-month ASAS − mean baseline ASAS)*100/baseline ASAS; this metric allows for a standardized comparison even if the baseline values are different between groups (IBM SPSS Statistics 29.0).

## 3 Results

### 3.1 Pre-SDR (baseline) participants’ characteristics

**Figure 1:**
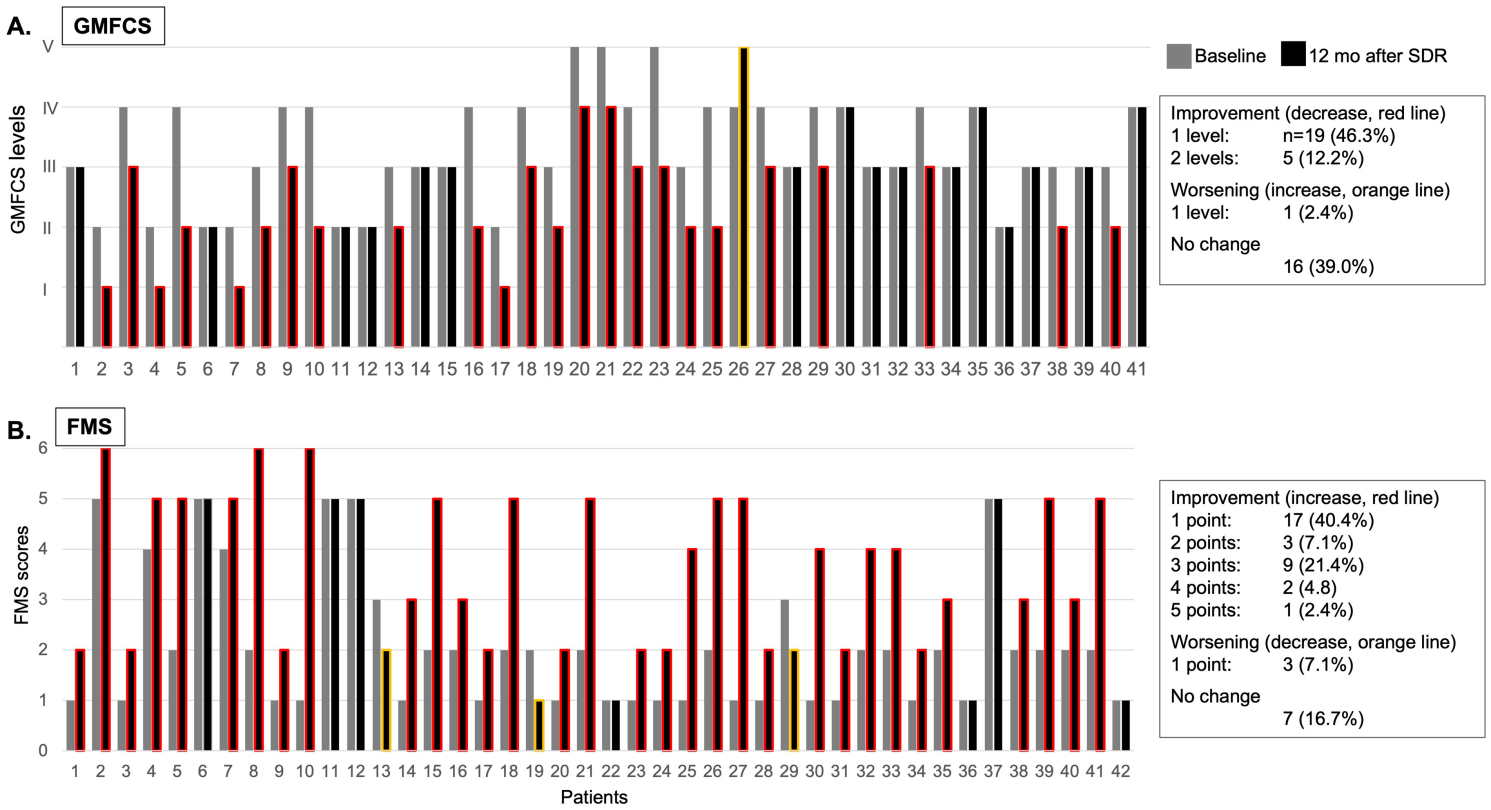
Individual GMFCS levels **(A)** and FMS scores **(B)** at baseline (grey column) and at 12 months after (black column) selective dorsal rhizotomy (SDR; red line signifies improvement, orange line signifies decline, no line signifies no change).

**Figure 2:**
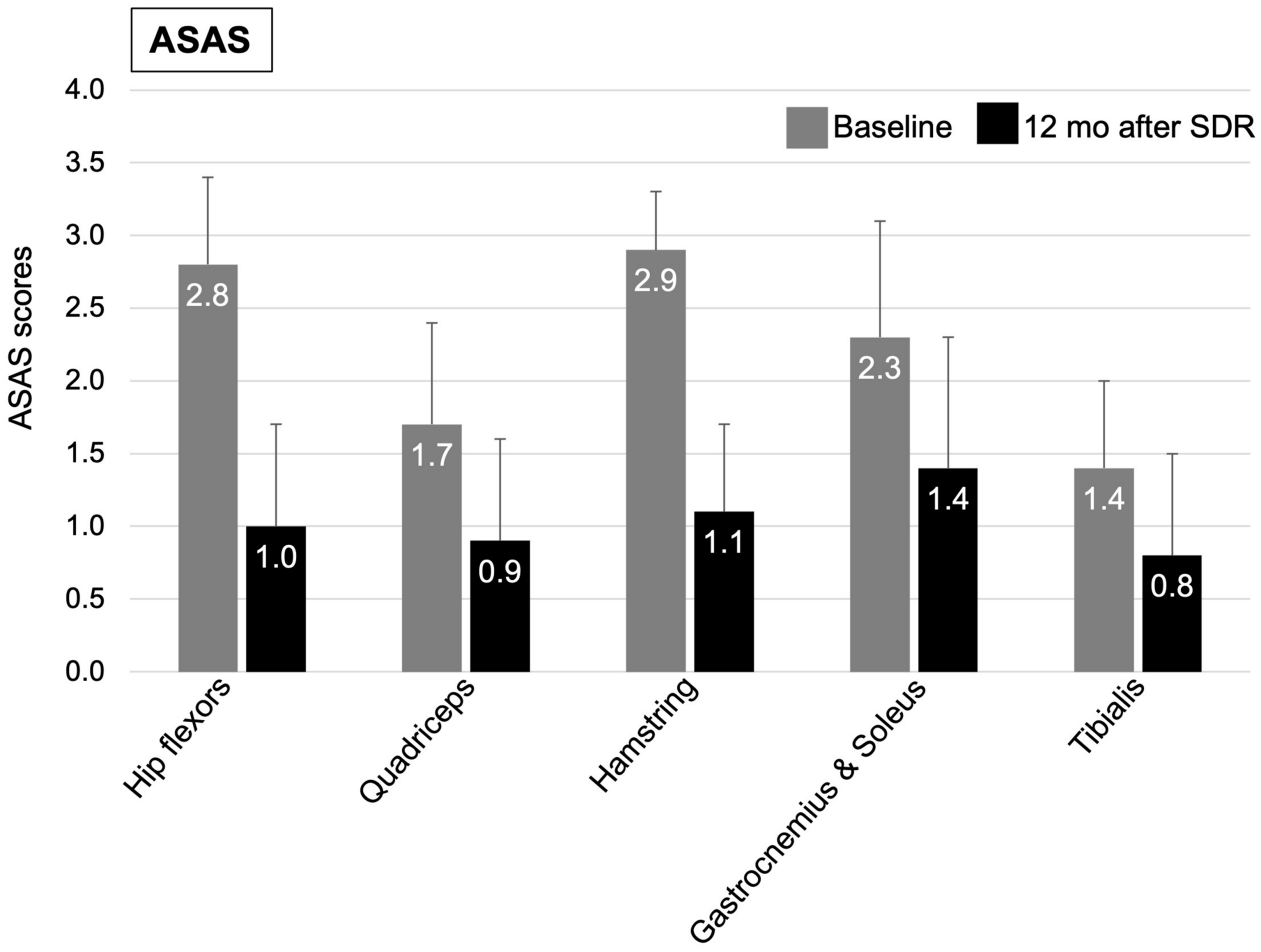
Mean (+SD) of ASAS clinical scores at baseline (grey column) and at 12 months after (black column) selective dorsal rhizotomy.

Data for 55 participants were extracted, but only 43 patients had completed the 12-month clinical follow-up and included (Table 1; 65.1% boys, 34.9% girls; mean ± SD age at surgery, 6.2 ± 2.5 years; 67.4% with diplegia, 30.2% with quadriplegia, and 2.3% with hemiplegia; 97.7% bilateral deficits). All patients were diagnosed by the age of 2 years of age; 79.1% of cases developed SCP secondary to perinatal complications associated with premature birth, 9.3% of unknown prenatal etiology (having an uneventful pregnancy and birth), 7.0% due to perinatal asphyxia, 2.3% due to ischemic infarction of the middle cerebral artery, and 2.3% due to brain hemorrhage. Patients presented with moderate to severe spasticity in hip flexors (ASAS, 2.8 ± 0.6), hamstring (2.9 ± 0.4), and gastrocnemius and soleus (2.3 ± 0.8), and mild to moderate spasticity in quadriceps (1.7 ± 0.7) and tibialis (1.4 ± 0.6) and they walked using handheld mobility devices in most settings and wheeled mobility when traveling long distances or require physical assistance or powered mobility [GMFCS, III (III, IV); FMS, 2.0 (1.0, 2.0)] (Figures 1, 2 and Table 2). All patients (100%) had received physical therapy and Botox injections; none underwent orthopedic surgery prior to SDR.

**Table 1:** Baseline (before the SDR procedure) demographic and clinical characteristics of SCP participants (mean ± standard deviation and *n*, number; B, boys; G, girls; MCA, middle cerebral artery).

Patient characteristics	
Age (years)	6.2 ± 2.5 (3–12)
Sex	28 B/15 G
SCP diagnosis	
Diplegia	29
Quadriplegia	13
Hemiplegia	1
SDR type	
Bilateral	42
Unilateral	1
SCP etiology	
Premature birth	34
Prenatal (unknown)	4
Asphyxia	3
MCA infarction	1
Brain hemorrhage	1

**Table 2:** Baseline and 12-month GMFCS distribution [how many improved (I), worsened (W), or did not change (=); 1l = 1 level, 2l = 2 levels] and FMS (25th–75th percentile), *p*-value and *W*-test for 12-month vs. baseline comparisons in the entire sample.

GMFCS				
Levels	Baseline	12 months		
		↓ (I)	↑ (W)	=
I	0	0	0	0
II	8	4 (1l)	0	4
III	15	6 (1l)	0	9
IV	15	7 (1l) 4 (2l)	1 (1l)	3
V	3	2 (1l) 1 (2l)	0	0
FMS				
Baseline		12 months		
2.0 (1.0, 2.0)		3.5 (2.0, 5.0)		
*p* < 0.001, *W* = 598.5					

### 3.2 Functional mobility significantly improved at 12 months after SDR

At 12 months after SDR, 58.5% patients demonstrated a significant improvement in GMFCS levels, 46.3% improved with one level, 12.2% improved with two levels, while 39.0% remained at their pre-SDR level, and 2.4% worsened with one level (Figure 1A and Table 2). Most patients (76.2%) improved FMS, 16.7% remained stable (or no change), and few showed a decline (7.1%) [for the entire sample, 3.5 (2.0, 5.0), *p* < 0.001, test statistic *W* = 598.5; Figure 1B and Table 2]. A significant decrease in the spasticity scores was reported for all muscles (hip flexors, 1.0 ± 0.7, % change, −62.7%; quadriceps, 0.9 ± 0.7, −46.0%; hamstring, 1.1 ± 0.5, −62.7%; gastrocnemius and soleus, 1.4 ± 0.8, −41.9%; tibialis, 0.8 ± 0.7, −43.1%; *p* < 0.001 for all, *t* varied from 4.7 to 19.9; Figure 2). These findings, taken together, suggest a significant improvement in functional mobility and less assistance 12 months after the SDR procedure.

### 3.3 Twelve-month improvement is more evident in younger patients

An analysis of age-related subgroups (<10 years old vs. ≥10 years old) demonstrated that 46.9% of younger patients improved the GMFCS with at least one level vs. none in older participants (Table 3). The FMS scores improved significantly in younger (*p* < 0.001, *W* = 268.5) compared to older (*p* = 0.6, *W* = 4.0); correlation analysis also demonstrated significant relationships between age at surgery and Δ FMS (*r* = 0.36, *p* = 0.02). These results suggest that younger children exhibited the largest improvement in functional mobility and assistance at 12 months after the SDR procedure compared to older children.

**Table 3:** Baseline and 12-month GMFCS distribution [how many improved (I), worsened (W), or did not change (=); 1l = 1 level, 2l = 2 levels] and FMS (25th–75th percentile), *p*-value and *W*-test for 12-month vs. baseline comparisons in age subgroups (>10 vs. <10 years old at the time of surgery).

>10 years old					<10 years old			
GMFCS								
Levels	Baseline	12 months			Baseline	12 months		
		↓ (I)	↑ (W)	=		↓ (I)	↑ (W)	=
I	0	0	0	0	0	0	0	0
II	3	0	0	3	5	2 (1l)	0	3
III	1	0	0	1	13	4 (1l)	0	9
IV	1	0	0	1	11	7 (1l)	1	3
V	0	0	0	0	3	2	0	1
FMS								
Baseline		12 months			Baseline		12 months	
3.5 (1.5, 4.7)		4.0 (2.3, 5.0)			2.0 (1.0, 2.0)		3.5 (2.0, 5.0)	
*p* = 0.6, *W* = 4.0					*p* < 0.001, *W* = 268.5			

### 3.4 Twelve-month improvement is similar between girls and boys

A similar subgroup analysis, this time in boys and girls, demonstrated improvement in GMFCS levels in a similar number of patients per subgroup (44.0% of boys vs. 37.5% of girls). Both subgroups significantly improved FMS scores (*p* < 0.001 in boys vs. *p* = 0.04 in girls), although the magnitude of improvement was higher in boys (Table 4).

**Table 4:** Baseline and 12-month GMFCS distribution [how many improved (I), worsened (W), or did not change (=); 1l = 1 level, 2l = 2 levels] and FMS (25th–75th percentile), *p*-value and *W*-test for 12-month vs. baseline comparisons in sex subgroups (boys vs. girls).

Boys					Girls			
GMFCS								
Levels	Baseline	12 months			Baseline	12 months		
		↓ (I)	↑ (W)	=		↓ (I)	↑ (W)	=
I	0	0	0	0	0	0	0	0
II	3	2 (1l)	0	1	5	0	0	5
III	11	2 (1l)	0	9	4	2 (1l)	0	2
IV	8	5	0	3	6	4 (1l)	1	1
V	3	2	0	1	0	0	0	0
FMS								
Baseline		12 months			Baseline		12 months	
1.5 (1.0, 2.0)		2.5 (2.0, 5.0)			2.0 (1.3, 4.8)		5.0 (3.3, 5.0)	
*p* < 0.001, *W* = 301.0					*p* = 0.04, *W* = 55.0			

### 3.5 Twelve-month improvement is more evident in severely impaired patients

A subgroup analysis, based on baseline clinical impairment, suggested that the best candidates for this procedure are those exhibiting severe functional impairment (GMFCS levels IV–V) at baseline (12-month improvement in 77.7% of patients compared to 43.5% of those with levels I–III, Table 5). Yet, considering baseline FMS scores, only those with severe impairment at baseline exhibited a significant improvement at follow-up (*p* < 0.001, *W* = 501.0; Table 5).

**Table 5:** Baseline and 12-month GMFCS distribution [how many improved (I), worsened (W), or did not change (=); 1l = 1 level, 2l = 2 levels] and FMS (25th–75th percentile), *p*-value and *W*-test for 12-month vs. baseline comparisons in clinical subgroups (mild-to-moderate vs. severe impairment).

Mild-to-moderate impairment					Severe impairment				
GMFCS									
Levels	Baseline	12 months			Levels	Baseline	12 months		
		↓ (I)	↑ (W)	=			↓ (I)	↑ (W)	=
I	0	0	0	0	IV	15	7 (1l) 4 (2l)	1 (1l)	3
II	8	4 (1l)	0	4	V	3	2 (1l) 1 (2l)	0	0
III	15	6 (1l)	0	9					
FMS									
Levels	Baseline		12 months		Levels	Baseline		12 months	
4–6	5.0 (4.5, 5.0)		5.0 (5.0, 5.0)		1–3	1.0 (1.0, 2.0)		3.0 (2.0, 5.0)	
*p* = 0.08, *W* = 6.0					*p* < 0.001, *W* = 501.0				

### 3.6 Post-SDR complications

After the SDR procedure, two patients developed the cerebrospinal fluid leak; one required surgical exploration and underwent dural defect closure and the other patient underwent skin suture at the bedside. One patient developed permanent loss of sensation in the right lower extremity in L5 dermatomal distribution. The remaining 40 patients have not reported or experienced SDR-related adverse events.

### 3.7 Twelve months post-SDR medical and surgical management

Compared to pre-SDR management, all patients continued physiotherapy, less received Botox (by 97.7%), and more received adjunct orthopedic surgery (32.6% vs. none) after SDR. An analysis of those who underwent vs. no orthopedic surgery did not reveal significant differences in age, sex, or baseline clinical impairment (*p* > 0.05 for all). Half of those receiving post-SDR adjuvant surgeries improved GMFCS (decreased by one level) compared to those without (64.0% improved with one to two levels) at 12 months after SDR; both subgroups improved significantly FMS scores (Table 6).

**Table 6:** Baseline and 12-month GMFCS distribution [how many improved (I), worsened (W), or did not change (=); 1l = 1 level, 2l = 2 levels] and FMS (25th–75th percentile), *p*-value and *W*-test for 12-month vs. baseline comparisons in post-SDR surgeries subgroups (surgeries vs. no surgeries).

Post-SDR surgeries					No post-SDR surgeries			
GMFCS								
Levels	Baseline	12 months			Baseline	12 months		
		↓ (I)	↑ (W)	=		↓ (I)	↑ (W)	=
I	0	0	0	0	0	0	0	0
II	3	2 (1l)	0	1	5	2 (1l)	0	3
III	9	3 (1l)	0	6	6	3 (1l)	0	3
IV	3	2 (1l)	0	1	12	5 (1l) 4 (l2)	1 (1l)	2
V	1	1 (1l)	0	0	2	1 (1l) 1 (2l)	0	0
FMS								
Baseline		12 months			Baseline		12 months	
2.0 (1.0, 2.0)		3.0 (2.0, 5.0)			2.0 (1.0, 2.0)		4.0 (2.0, 5.0)	
*p* = 0.002, *W* = 99.5					*p* < 0.001, *W* = 220.0			

## 4 Discussion

This study is the first to examine the long-term functional outcomes of SDR in pediatric patients with SCP in Jordan. Our findings show notable improvement in gross motor skills and functional mobility, as well as a decrease in muscle stiffness in all studied muscle groups at 12 months after SDR, illustrating the beneficial effects of SDR on functional mobility of children with SCP.

The changes in GMFCS levels and FMS scores suggest that SDR successfully boosts gross motor function and functional mobility (Figure 1 and Table 2). The decrease in GMFCS levels (with one to two levels) in 58.5% of patients and a significant increase in FMS scores in 76.2% demonstrate improvement in patients’ mobility and capacity to engage in daily activities with less assistance. Notably, the decrease in muscle spasticity, specifically in the hip flexors (by 62.8%) and hamstrings (by 61.2%), underscores the impact of the SDR on these crucial muscle groups for walking and movement. These results are consistent with prior findings in the U.S. and Germany that have demonstrated the efficacy of SDR in decreasing spasticity and improving motor function in similar samples (12, 13).

Our results also showed noteworthy effects of age at surgery on GMFCS levels and FMS scores, indicating that younger patients may experience greater improvements in gross motor function and assistance from the procedure (Table 3). Specifically, 46.9% of patients younger than 10 years at the time of surgery had the greatest functional outcomes as suggested by decreased GMFCS levels and increased FMS scores compared to those older than 10 years (no patient improved GMFCS level and no significant changes in FMS, Table 3). These findings highlight the need for timely intervention in improving results for SCP patients undergoing SDR; older patients might see less significant enhancements in functional mobility with SDR, possibly due to ingrained movement patterns and physical deformities secondary to spasticity. This is consistent with prior evidence indicating a similar decrease in SDR benefits in patients older than 10 years (14, 15). If larger studies demonstrate a similar age-dependent SDR benefit, this would be crucial in identifying the best therapeutic window for this procedure in Jordan (and possibly globally). Therefore, if the goal of treatment is directed toward improving functional mobility in patients older than 10 years, then the option of SDR would not be recommended in this sample. As Al-Otaibi et al. (16) suggested, it is imperative to identify the treatment goals to set up appropriate expectations and decide the best next steps for each case; if the goal is to improve quality of life by decreasing pain, then this procedure could be appropriate in patients older than 10 years.

Our results also showed that patients with severe neurological impairment at baseline experienced greater improvements in gross motor function and, particularly, in functional mobility after the SDR procedure compared to those mildly impaired (Table 5). These findings are consistent with previous studies showing better SDR outcomes in those with higher pre-SDR GMFCS scores (more impaired) (15, 17) and suggest the importance of functional mobility impairment (in addition to the age at surgery) when determining the best candidates for SDR. Further work with larger sample sizes is warranted to determine eligibility for the different age and functional mobility subgroups.

Further, the ongoing utilization of physical therapy after SDR, along with additional orthopedic surgeries, highlights the need for a multidisciplinary approach to treating SCP even after SDR (5). In general, the need for additional orthopedic interventions, like tendon release or lengthening, after the SDR procedure depends on the severity of spasticity and functional impairment; these interventions lead to superior functional recovery (5, 18) and a decreased need for lower extremity bracing (19) in these patients. In our cohort, those requiring such interventions post-SDR have had soft tissue deformities or muscle shortening and represented 32.6% of our cohort (Table 6); these data corroborate well prior findings demonstrating a possible need for future orthopedic surgeries after SDR in a similar population (20). Moreover, SDR does not significantly affect the need for orthopedic procedures involving permanent bone deformities (e.g., ankle/foot corrections and femoral osteotomies) (20). Taken together, it is likely that SDR, along with other adjunct therapies, works synergistically in contributing to the outcomes; this reinforces the need for a control group (no SDR) for comparison.

The current study has several limitations. The first limitation is the retrospective design; a prospective study would provide a fuller picture of the effects of this procedure not only on functional mobility and spasticity scores but also on quality of life and other symptoms (e.g., pain). Second, the lack of a control group undergoing no SDR procedure limits our interpretation. Third, the small sample size and short follow-up period (12 months) also limit the ability to capture the long-term effects, years to decades, of this procedure. Fourth, because we did not evaluate clinical status after the SDR procedure, the surgical/non-surgical treatments performed after the SDR procedure may contribute, at least partially, to the 12-month improvement reported here. Yet, our data revealed that those who received post-SDR orthopedic surgery improved less than those without such interventions. Future studies in Jordan should consider a prospective design with a larger sample size, immediate post-SDR clinical evaluations, and extended follow-up periods to examine the impact of SDR on function and quality of life and possibly identify any long-term adverse effects. Fifth, reported FMS changes here reached statistical significance but may not be clinically meaningful; since the minimum clinically important differences for this scale had not been established in this population, these data should be interpreted cautiously. Sixth, it is necessary to account for other commonly seen CP-associated factors, like visual and speech impairments (21, 22), which are not included here. Another factor to be considered in future studies is the socioeconomic status, which can significantly impact care delivery and decrease their ability to follow up; this is especially true in developing countries like Jordan (21, 22).

In conclusion, our results demonstrated the benefit of the SDR procedure to decrease spasticity and enhance functional mobility in children with SCP in Jordan, emphasizing the significance of early surgical treatment and providing future support for the ongoing use and further exploration of SDR as a standard treatment for SCP in children; these data confirm prior SDR findings in this population (6–8). Our results also suggest the importance of collaborative multidisciplinary teams to provide services and resources based on patient needs, suggesting the necessity for a holistic, team-based approach to enhance patient results. Further, the knowledge acquired from this research may help healthcare providers to better educate and advise patients and their families on the possible advantages and constraints of the SDR procedure, ultimately leading to an enhanced quality of life for children with SCP.

## Data Availability

The raw data supporting the conclusions of this article will be made available by the authors, without undue reservation.
